# Supplementary material for: Time-resolved crystallography of boric acid binding to the active site serine of the β-lactamase CTX-M-14 and subsequent 1,2-diol esterification
Source: Commun Chem. 2024 Jul 5;7:152. doi: 10.1038/s42004-024-01236-w (PMC11226702; doi:10.1038/s42004-024-01236-w)
Supplement: Supplementary file 2 — Supplementary Information [file 42004_2024_1236_MOESM2_ESM.pdf]

Supplementary Information

Time-resolved crystallography of boric acid binding to the active site serine of the β-lactamase CTX-M-14 and subsequent 1,2-diol esterification

Andreas Prester[1,2], Markus Perbandt[3], Marina Galchenkova[4], Dominik Oberthuer[4], Nadine Werner[3,5], Alessandra Henkel[4], Julia Maracke[4], Oleksandr Yefanov[4], Johanna Hakanpää[6], Guillaume Pompidor[6], Jan Meyer[6], Henry Chapman[4,7,8], Martin Aepfelbacher[1], Winfried Hinrichs[9], Holger Rohde[1,*] and Christian Betzel[3,7,*]

[1] Institute of Medical Microbiology, Virology, and Hygiene, University Medical Center Hamburg-Eppendorf UKE, Hamburg, Germany.

[2] Present address: Institute of Biochemistry and Signal Transduction, University Medical Center Hamburg-Eppendorf UKE, Hamburg, Germany.

[3] Institute of Biochemistry and Molecular Biology, University of Hamburg, Hamburg, Germany.

[4] Center for Free-Electron Laser Science CFEL, DESY, Hamburg, Germany.

[5] Present address: Centre for Integrative Biology, Department of Integrated Structural Biology, Institute of Genetics and of Molecular and Cellular Biology IGBMC, Illkirch, France.

[6] Deutsches Elektronen-Synchrotron DESY, Hamburg, Germany

[7] Hamburg Centre for Ultrafast Imaging CUI, University of Hamburg, Hamburg, Germany

[8] Department of Physics, University of Hamburg, Hamburg, Germany

[9] Institute of Biochemistry, University of Greifswald, Greifswald, Germany.

*Corresponding authors: Christian Betzel (christian.betzel@uni-hamburg.de) and Holger Rohde (rohde@uke.de)

Abstract

The emergence and spread of antibiotic resistance represent a growing threat to public health. Of particular concern is the appearance of β-lactamases, which are capable to hydrolyze and inactivate the most important class of antibiotics, the β-lactams. Effective β-lactamase inhibitors and mechanistic insights into their action are central in overcoming this type of resistance, and in this context boronate-based β-lactamase inhibitors were just recently approved to treat multidrug-resistant bacteria. Using boric acid as a simplified inhibitor model, time-resolved serial crystallography was employed to obtain mechanistic insights into binding to the active site serine of β-lactamase CTX-M-14, identifying a reaction time frame of 80 – 100 ms. In a next step, the subsequent 1,2-diol boric ester formation with glycerol in the active site was monitored proceeding in a time frame of 100 – 150 ms. Furthermore, the displacement of the crucial anion in the active site of the β-lactamase was verified as an essential part of the binding mechanism of substrates and inhibitors. In total, 22 datasets of β-lactamase intermediate complexes with high spatial resolution of 1.40 – 2.04 Å and high temporal resolution range of 50 – 10000 ms were obtained, allowing a detailed analysis of the studied processes. Mechanistic details captured here contribute to the understanding of molecular processes and their time frames in enzymatic reactions. Moreover, we could demonstrate that time-resolved crystallography can serve as an additional tool for identifying and investigating enzymatic reactions.

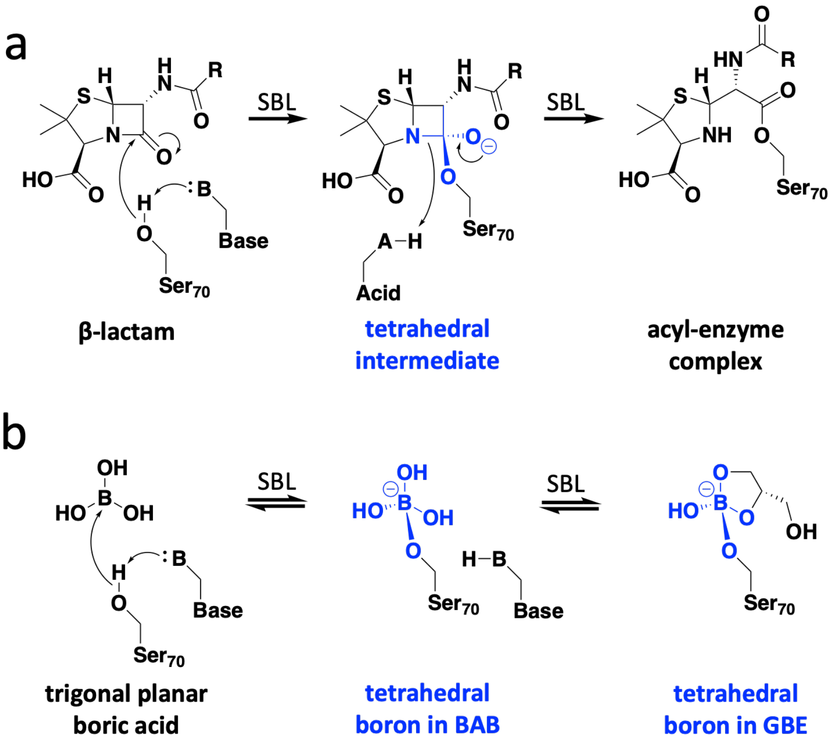

Supplementary Figure 1. Comparison of substrate acylation to boric acid and glycerol boric acid ester binding to serine β-lactamases. Common tetrahedral intermediate before acyl-enzyme complex formation during enzymatic β-lactam hydrolysis by serine-β-lactamases (SBL; a). A general base subtracts the proton from Ser70 OG, activating it for a nucleophilic attack on the amide carbonyl carbon atom of the β-lactam ring, resulting in a tetrahedral acylation transition state (blue). The transition state is resolved by the formation of a covalent acyl-enzyme intermediate by a general acid that donates a proton to the β lactam ring nitrogen. In comparison, there is the stable transition state analog with the tetrahedral boron atom in CTX-M-14 complexed with BAB or GBE (b). Binding of boric acid also involves a nucleophilic attack of Ser70 OG after its activation by a general base.

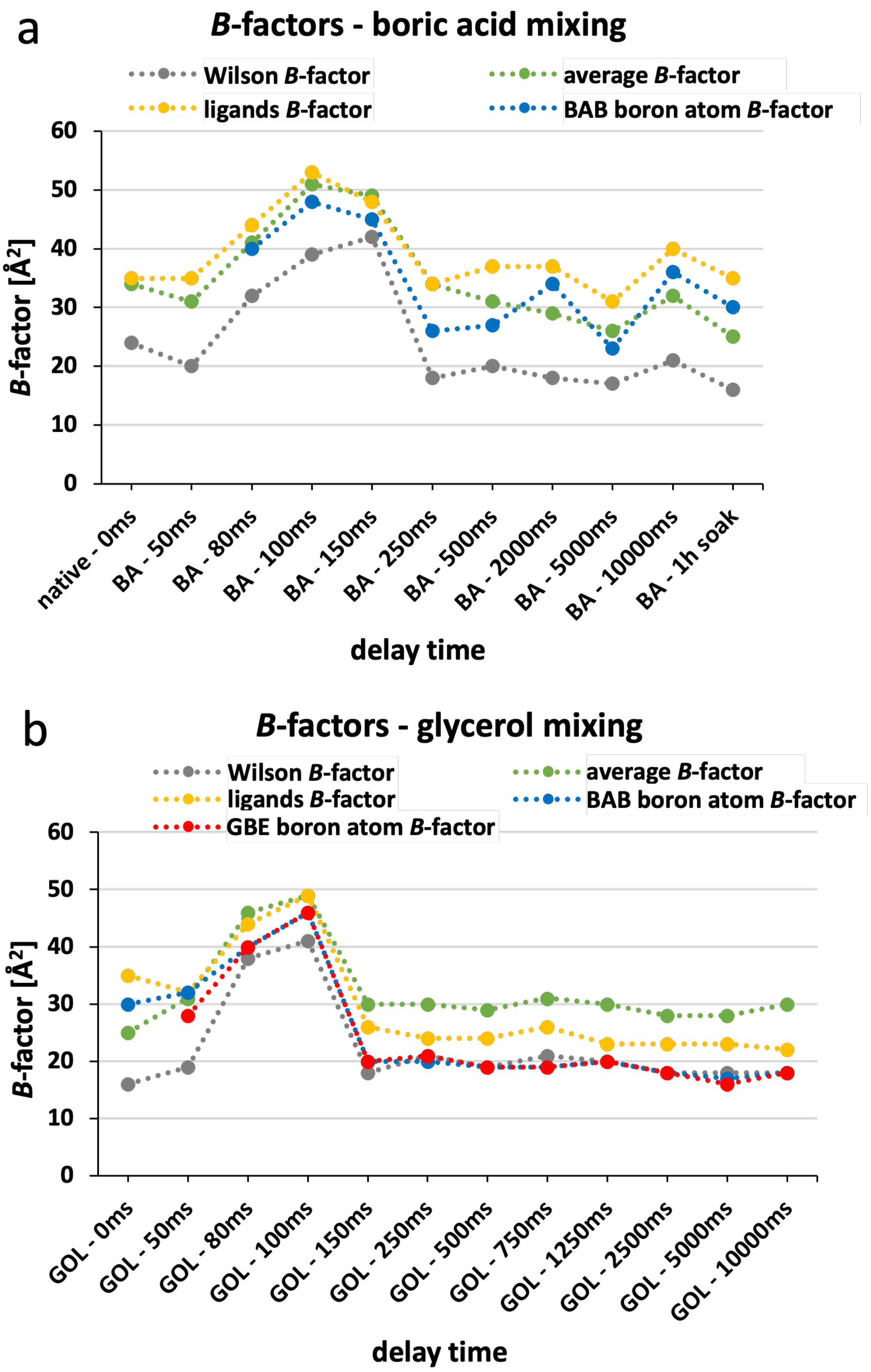

Supplementary Figure 2. Progression of *B*-factors in CTX-M-14 structures with bound boric acid and glycerol boric acid ester during the observed time frame in time-resolved crystallography experiments. Diagram of the refined atomic *B*-factors of the boron atom of bound boric acid (a, BAB) and the glycerol boric acid ester (b, GBE) as a function of the respective mixing delay times with boric acid (BA) or subsequent glycerol (GOL) mixing. *B*-Factor ligands contains the *B*-factors of all ligands present in the respective structure.

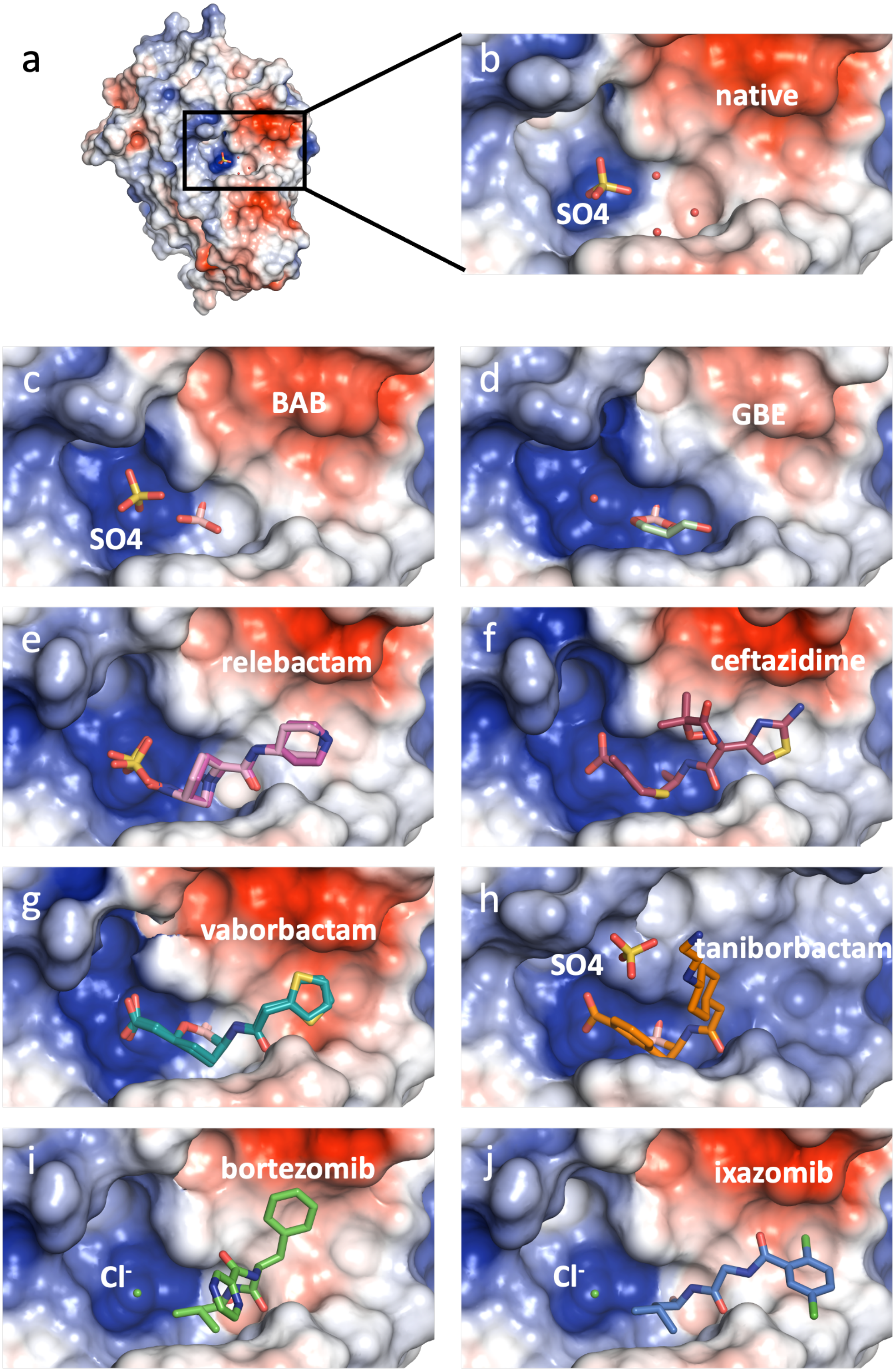

Supplementary Figure 3. Electrostatic surface representation of β-lactamase active site with various inhibitors and substrates. (a) CTX-M-14 native enzyme overview**, (b)** CTX-M-14 native enzyme

(pdb:8pc9), **(c)** CTX-M-14 in complex with bound boric acid (pink/red, 10s BAB, 8pcj), **(d)** CTX-M-14 in complex with bound glycerol boric acid ester (pale green, 10s GBE, 8pcv), **(e)** CTX-M-14 in complex with bound relebactam (pink, unpublished), **(f)** CTX-M-14 E166A mutant variant in complex with bound ceftazidime (raspberry, 5u53[1]), **(g)** CTX-M-14 in complex with bound vaborbactam (teal, 6v7h[2]), **(h)** CTX-M-15 in complex with bound taniborbactam (orange, 6sp6[3]), **(i)** CTX-M-14 in complex with bound bortezomib (green, 7q0y[4]), **(j)** CTX-M-14 in complex with bound ixazomib (blue, 7q11[4]). Positively charged areas of the enzyme surface are displayed in blue and negatively charged areas in red. The binding pocket of CTX-M-14 forms a well-defined positively charged region, also referred to as the anion binding site, which is utilized by negative charged groups of several inhibitors and substrates for stronger binding. The electrostatic surface representations were calculated and visualized applying the APBS Electrostatics plugin in PyMOL[5,6].

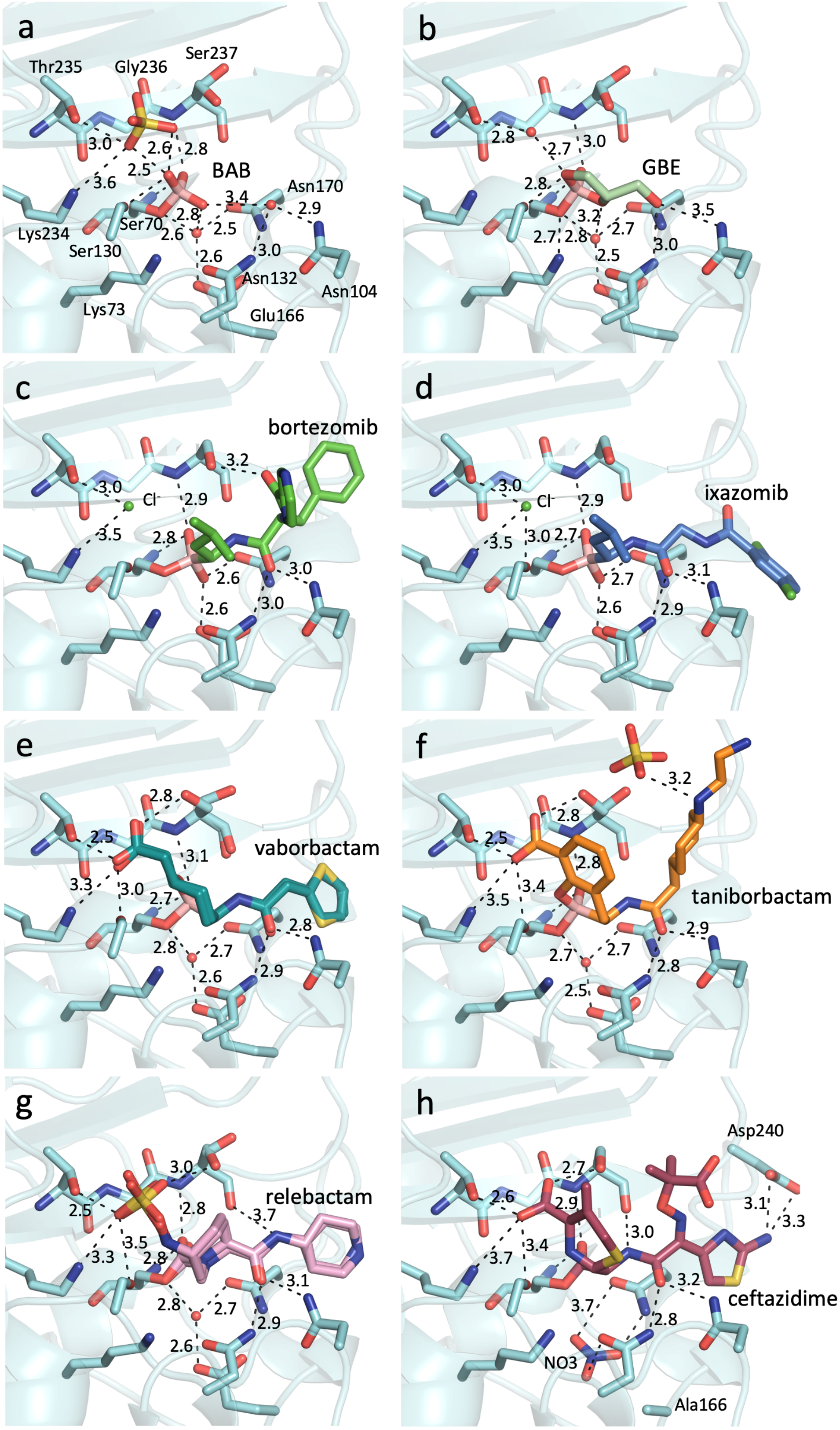

Supplementary Figure 4. Comparison of β-lactamase active sites with various inhibitors and substrates for the analysis to identify advantageous features to be considered for the development of β-lactamase inhibitors. CTX-M-14 in complex with **(a)** bound boric acid (pink/red, 10s BAB, 8pcj),

(b) bound glycerol boric acid ester (pale green, 10s GBE, 8pcv), **(c)** bound bortezomib (green, 7q0y[4]), **(d)** bound ixazomib (blue, 7q11[4]), **(e)** bound vaborbactam (teal, 6v7h[2]). **(f)** CTX-M-15 in complex with bound taniborbactam (orange, 6sp6[3]), **(g)** CTX-M-14 in complex with bound relebactam (pink, unpublished), and **(h)** CTX-M-14 E166A mutant in complex with bound ceftazidime (raspberry, 5u53[1]). Potential hydrogen bond distances (Å) are indicated by dashed lines.

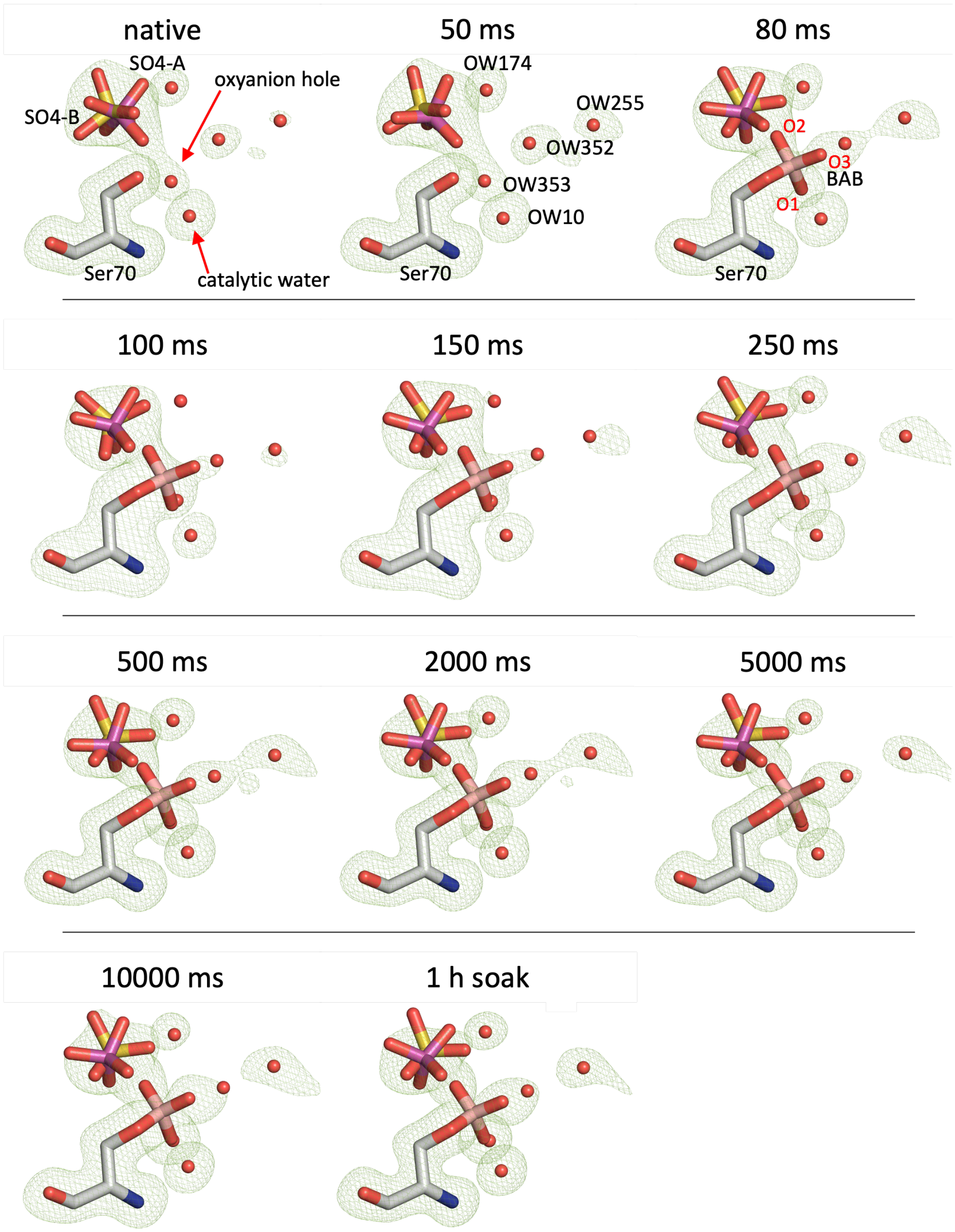

Supplementary Figure 5. Full timeline of the active site of CTX-M-14 mixed with boric acid at delay times of 50 ms – 10000 ms. Polder electron density (contoured at 5 σ, green mesh) of the active site Ser70, the sulfate ions and bound boric acid (BAB) are shown at different delay time points after mixing microcrystals with boric acid. The 1 h soak structure was obtained with the TapeDrive after microcrystals have been soaked in boric acid for 1 h and shows that almost no further increase in electron density is observed after 10 s.

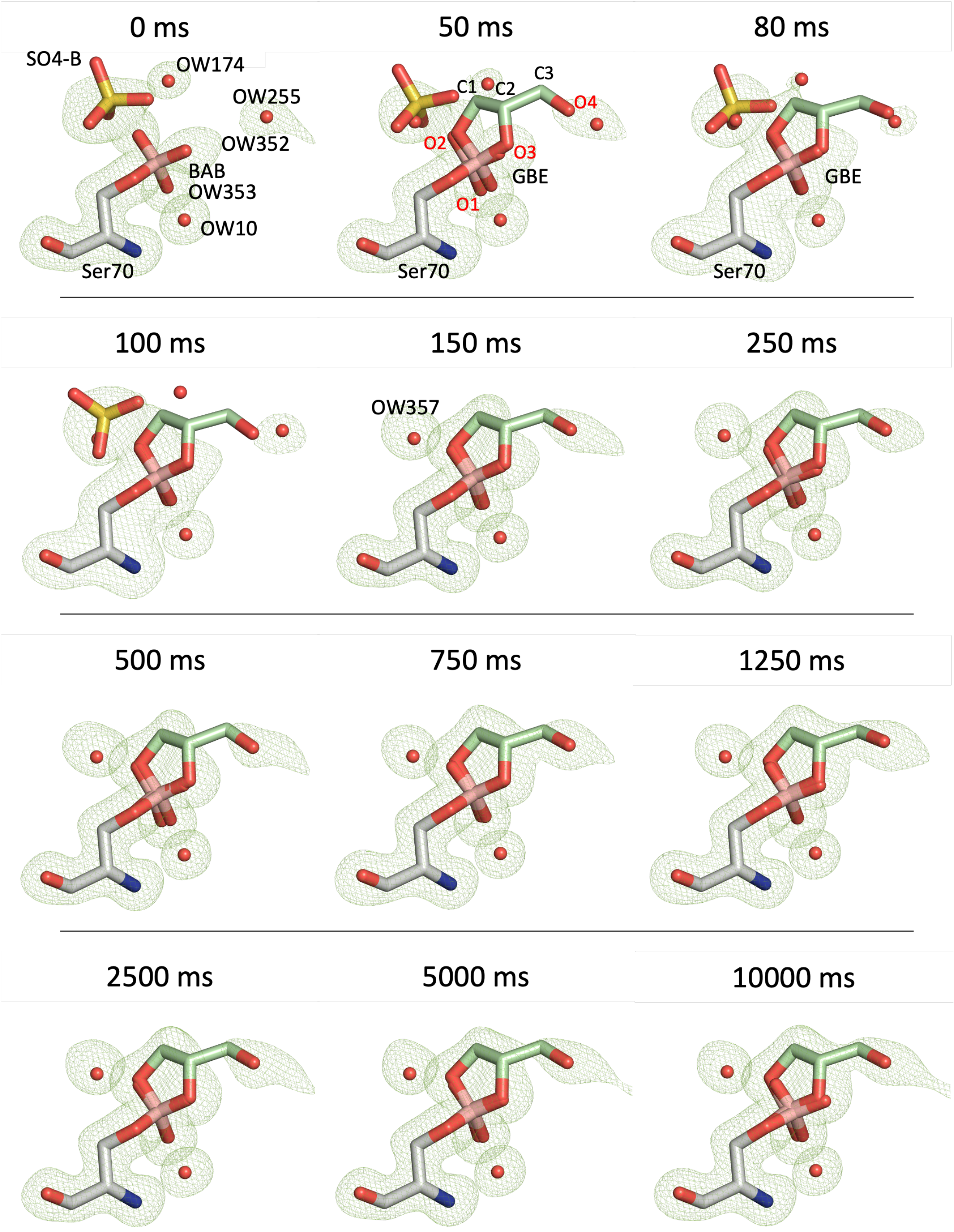

Supplementary Figure 6. Full timeline of the active site of CTX-M-14 presoaked with boric acid and subsequently mixed with glycerol at delay times of 50 ms – 10000 ms applying the TapeDrive. Polder electron densities (contoured at 5 σ, green mesh) of the active site region of CTX-M-14. Time-resolved observation of the ester bond formation between glycerol and the Ser70 borate ester. The sulfate anion present in the native conformation is displaced upon binding of GBE and finally replaced by solvent water OW357.

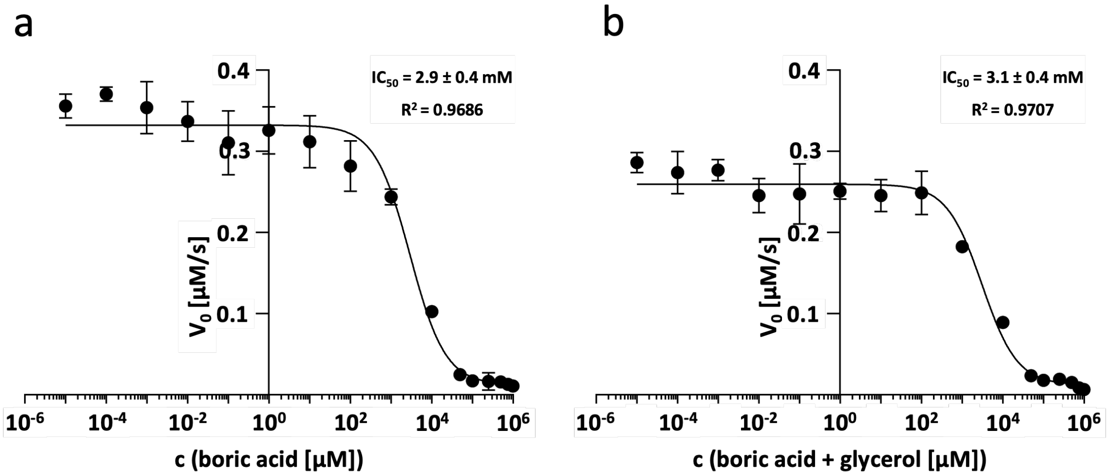

Supplementary Figure 7. Determination of the IC_{50} values applying boric acid and a combination of boric acid and glycerol as an inhibitor. The initial velocity rates of CTX-M-14 after 15 minutes pre-incubation with the inhibiting compound at varying concentrations were monitored following the hydrolysis of 100 µM cefotaxime and resulted in IC_{50} values of 2.9 ± 0.4 mM for boric acid (a) and 3.1 ± 0.4 mM for boric acid with glycerol (b). Data points are shown as mean values with standard deviation error bars.

Supplementary Table 1: Serial diffraction data collection and refinement statistics for CTX-M-14 β-lactamase mixing with boric acid (BAB).

	CTX-M-14 native 0 ms	CTX-M-14 BAB 50 ms	CTX-M-14 BAB 80 ms	CTX-M-14 BAB 100 ms	CTX-M-14 BAB 150 ms	CTX-M-14 BAB 250 ms	CTX-M-14 BAB 500 ms	CTX-M-14 BAB 2000 ms	CTX-M-14 BAB 5000 ms	CTX-M-14 BAB 10000 ms
PDB entry	8pc9	8pca	8pcb	8pcc	8pcd	8pce	8pcf	8pcg	8pci	8pcj
Resolution range (Å)	17.64-1.40 (1.42-1.40)	38.88-1.58 (1.60-1.58)	38.88-1.69 (1.71-1.69)	38.88-2.04 (2.06-2.04)	38.88-1.97 (1.99-1.97)	38.88-1.65 (1.67-1.65)	38.88-1.50 (1.52-1.50)	38.88-1.51 (1.53-1.51)	38.88-1.48 (1.50-1.48)	38.88-1.65 (1.67-1.65)
Space group	P3221	P3221	P3221	P3221	P3221	P3221	P3221	P3221	P3221	P3221
a, b, c, (Å)	42.10, 42.10, 234.47	41.84, 41.84, 233.28	41.84, 41.84, 233.28	41.84, 41.84, 233.28	41.84, 41.84, 233.28	41.84, 41.84, 233.28	41.84, 41.84, 233.28	41.84, 41.84, 233.28	41.84, 41.84, 233.28	41.84, 41.84, 233.28
α, β, γ (°)	90, 90, 120	90, 90, 120	90, 90, 120	90, 90, 120	90, 90, 120	90, 90, 120	90, 90, 120	90, 90, 120	90, 90, 120	90, 90, 120
Unique reflections	48378 (3119)	33953 (2151)	27958 (1837)	16186 (1063)	17902 (1167)	29970 (1934)	39474 (2460)	38753 (2453)	40899 (2400)	29963 (1928)
⟨I/σ(I)⟩	9.09 (0.72)	30.27 (0.26)	16.44 (0.46)	7.17 (1.11)	9.17 (0.44)	18.97 (0.57)	26.93 (0.55)	24.60 (0.38)	23.33 (0.09)	26.86 (0.48)
Completeness (%)	100 (100)	99.77 (96.54)	100 (100)	100 (100)	100 (100)	100 (100)	99.74 (95.98)	99.91 (98.63)	99.36 (90.09)	99.98 (99.69)
Multiplicity	2214 (1363)	13328 (8.7)	7241 (1889.0)	3032 (1596.0)	3925 (1855.9)	15231 (26.9)	7186 (10.3)	7160 (11.2)	5842 (5.9)	8047 (13.2)
Rsplit	7.20 (157.58)	3.70 (272.32)	3.83 (225.30)	9.99 (104.11)	7.44 (250.08)	5.60 (188.20)	3.13 (232.82)	3.97 (241.59)	4.10 (225.50)	4.39 (243.74)
CC1/2	0.991 (0.273)	0.999 (0.171)	0.999 (0.154)	0.995 (0.188)	0.996 (0.170)	0.998 (0.227)	0.999 (0.191)	0.999 (0.158)	0.999 (0.155)	1.000 (0.164)
CC*	0.998 (0.655)	1.000 (0.541)	1.000 (0.516)	0.999 (0.563)	0.999 (0.539)	1.000 (0.609)	1.000 (0.567)	1.000 (0.522)	1.000 (0.518)	1.000 (0.531)
Wilson *B*-factor (Å²)	23.58	19.96	32.20	38.85	41.93	18.36	20.03	17.91	16.98	21.34
Resolution range in refinement (Å)	17.64-1.40 (1.45-1.40)	38.88-1.58 (1.64-1.58)	38.88-1.69 (1.75-1.69)	38.88-2.04 (2.11-2.04)	38.88-1.97 (2.04-1.97)	38.88-1.65 (1.71-1.65)	38.88-1.53 (1.59-1.53)	38.88-1.52 (1.56-1.52)	38.88-1.50 (1.55-1.50)	38.88-1.65 (1.71-1.65)
Reflections used in refinement	48059 (4652)	33822 (3036)	27707 (2665)	16067 (1562)	17763 (1706)	29817 (2887)	37250 (3656)	38607 (3539)	39413 (3784)	29844 (2830)
Reflections used for Rfree	2790 (271)	1956 (183)	1602 (150)	937 (89)	1027 (97)	1728 (165)	2159 (213)	2221 (202)	2284 (221)	1726 (162)
Rwork	0.145 (0.393)	0.144 (0.403)	0.152 (0.468)	0.188 (0.466)	0.171 (0.382)	0.163 (0.418)	0.135 (0.303)	0.142 (0.394)	0.143 (0.358)	0.142 (0.391)
Rfree	0.170 (0.399)	0.170 (0.398)	0.188 (0.455)	0.231 (0.498)	0.215 (0.455)	0.198 (0.448)	0.161 (0.299)	0.166 (0.418)	0.165 (0.359)	0.177 (0.433)
RMS bonds (Å)	0.009	0.008	0.005	0.003	0.004	0.007	0.009	0.010	0.017	0.012
RMS angles (°)	0.96	1.01	0.81	0.56	0.71	0.93	1.03	1.05	1.40	1.15
Ramachandran favored (%)	98.46	97.30	96.91	97.68	97.68	97.30	97.68	97.68	97.68	96.91
Ramachandran allowed (%)	1.16	2.32	2.70	1.93	1.93	2.32	1.93	1.93	1.93	2.70
Ramachandran outliers (%)	0.39	0.39	0.39	0.39	0.39	0.39	0.39	0.39	0.39	0.39
Rotamer outliers (%)	0.92	0.93	0.93	0.93	0.93	0.93	0.93	0.93	0.93	0.93
Clashscore	1.45	3.88	1.71	1.71	2.67	1.95	3.42	2.19	2.19	3.40
Average *B*-factor (Å²)	34.37	30.90	41.12	51.22	49.15	33.64	30.93	29.23	26.46	31.52
macromolecules (Å²)	32.65	29.21	39.71	50.70	48.32	32.12	29.05	27.42	25.69	29.74
ligands (Å²)	35.25	34.87	43.96	53.23	47.55	34.14	37.17	37.47	31.14	39.96
solvent (Å²)	49.23	44.92	53.03	56.68	56.20	45.97	45.62	43.44	35.61	45.57
MolProbity score	0.88	1.13	1.02	0.86	0.91	0.97	1.13	0.99	0.99	1.14

Statistics for the highest-resolution shell are shown in parentheses.

Supplementary Table 2: Serial diffraction data collection and refinement statistics for CTX-M-14 β-lactamase (presoaked with boric acid) mixing with glycerol (GBE).

	CTX-M-14 GBE 0 ms	CTX-M-14 GBE 50 ms	CTX-M-14 GBE 80 ms	CTX-M-14 GBE 100 ms	CTX-M-14 GBE 150 ms	CTX-M-14 GBE 250 ms	CTX-M-14 GBE 500 ms	CTX-M-14 GBE 750 ms	CTX-M-14 GBE 1250 ms	CTX-M-14 GBE 2500 ms	CTX-M-14 GBE 5000 ms	CTX-M-14 GBE 10000 ms
PDB entry	8pck	8pcl	8pcm	8pcn	8pco	8pcp	8pcq	8pcr	8pcs	8pct	8pcu	8pcv
Resolution range (Å)	35.81-1.51 (1.53-1.51)	38.88-1.60 (1.62-1.60)	38.88-1.84 (1.86-1.84)	38.88-1.90 (1.92-1.90)	38.88-1.55 (1.57-1.55)	36.23-1.76 (1.78-1.76)	38.88-1.52 (1.54-1.52)	35.81-1.70 (1.72-1.70)	35.81-1.67 (1.69-1.67)	38.88-1.53 (1.55-1.53)	38.88-1.50 (1.52-1.50)	38.88-1.58 (1.60-1.58)
Space group	$P3_221$	$P3_221$	$P3_221$	$P3_221$	$P3_221$	$P3_221$	$P3_221$	$P3_221$	$P3_221$	$P3_221$	$P3_221$	$P3_221$
a, b, c, (Å)	41.84, 41.84, 233.28	41.84, 41.84, 233.28	41.84, 41.84, 233.28	41.84, 41.84, 233.28	41.84, 41.84, 233.28	41.84, 41.84, 233.28	41.84, 41.84, 233.28	41.84, 41.84, 233.28	41.84, 41.84, 233.28	41.84, 41.84, 233.28	41.84, 41.84, 233.28	41.84, 41.84, 233.28
α, β, γ (°)	90, 90, 120	90, 90, 120	90, 90, 120	90, 90, 120	90, 90, 120	90, 90, 120	90, 90, 120	90, 90, 120	90, 90, 120	90, 90, 120	90, 90, 120	90, 90, 120
Unique reflections	38700 (2403)	32725 (2042)	21824 (1402)	19841 (1230)	35926 (2309)	24839 (1602)	38024 (2425)	27494 (1819)	28920 (1854)	37341 (2393)	39560 (2546)	34013 (2210)
⟨I/σ(I)⟩	10.43 (0.48)	23.76 (0.44)	10.73 (0.59)	10.65 (0.57)	26.85 (0.67)	10.52 (0.53)	18.55 (0.47)	10.72 (0.47)	11.29 (0.48)	16.31 (0.49)	27.45 (0.50)	17.47 (0.43)
Completeness (%)	99.79 (96.62)	99.88 (98.13)	100 (100)	100 (100)	99.93 (98.89)	100 (100)	99.80 (96.88)	100 (99.95)	100 (100)	99.86 (97.91)	99.96 (99.34)	99.95 (99.19)
Multiplicity	841 (7.0)	6417 (9.7)	3113 (1188.3)	3905 (1428.6)	10849(11.8)	1411 (20.5)	3101 (8.8)	1182 (24.6)	1149 (22.3)	2558 (9.5)	7437 (15.2)	3311 (10.1)
Rsplit	8.37 (221.50)	4.79 (295.84)	5.64 (178.63)	6.30 (189.92)	4.00 (229.50)	9.17 (204.95)	4.78 (235.48)	8.19 (234.06)	8.11 (235.34)	5.75 (225.93)	3.50 (216.25)	5.75 (239.54)
CC1/2	0.995 (0.175)	0.999 (0.187)	0.998 (0.195)	0.996 (0.199)	0.999 (0.181)	0.997 (0.225)	0.998 (0.218)	0.995 (0.161)	0.997 (0.150)	0.998 (0.161)	0.999 (0.174)	0.999 (0.200)
CC*	0.999 (0.546)	1.000 (0.561)	0.999 (0.572)	0.999 (0.577)	1.000 (0.553)	0.999 (0.606)	1.000 (0.598)	0.999 (0.526)	0.999 (0.510)	1.000 (0.527)	1.000 (0.544)	1.000 (0.577)
Wilson *B*-factor (Å²)	16.34	19.08	38.38	41.48	18.41	21.22	19.45	21.16	20.36	17.97	17.54	18.29
Resolution range in refinement (Å)	35.81-1.53 (1.59-1.53)	38.88-1.60 (1.66-1.60)	38.88-1.84 (1.91-1.84)	38.88-1.90 (1.97-1.90)	38.88-1.55 (1.61-1.55)	36.23-1.76 (1.82-1.76)	38.88-1.55 (1.61-1.55)	35.81-1.70 (1.76-1.70)	35.81-1.67 (1.73-1.67)	38.88-1.55 (1.61-1.55)	38.88-1.50 (1.55-1.50)	38.88-1.58 (1.63-1.58)
Reflections used in refinement	37258 (3616)	32612 (2965)	21655 (2123)	19688 (1843)	35787 (3360)	24717 (2419)	35817 (3501)	27370 (2672)	28807 (2791)	35828 (3497)	39421 (3757)	33900 (3184)
Reflections used for R_{free}	2166 (219)	1877 (170)	1251 (119)	1142 (107)	2067 (198)	1435 (142)	2078 (207)	1590 (154)	1669 (160)	2077 (207)	2286 (223)	1959 (186)
R_{work}	0.143 (0.359)	0.145 (0.394)	0.165 (0.369)	0.164 (0.420)	0.144 (0.380)	0.159 (0.360)	0.139 (0.345)	0.150 (0.353)	0.146 (0.367)	0.141 (0.352)	0.141 (0.368)	0.150 (0.377)
R_{free}	0.170 (0.361)	0.175 (0.404)	0.210 (0.372)	0.206 (0.468)	0.169 (0.375)	0.192 (0.327)	0.166 (0.363)	0.183 (0.395)	0.178 (0.391)	0.166 (0.359)	0.163 (0.383)	0.174 (0.348)
RMS bonds (Å)	0.013	0.007	0.003	0.004	0.012	0.005	0.008	0.004	0.006	0.017	0.014	0.008
RMS angles (°)	1.23	0.90	0.62	0.64	1.24	0.84	1.07	0.768	0.96	1.45	1.24	0.99
Ramachandran favored (%)	98.08	97.70	97.32	98.47	97.32	98.08	97.70	98.08	98.08	97.70	98.08	98.08
Ramachandran allowed (%)	1.53	1.92	2.30	1.15	2.30	1.53	1.92	1.53	1.53	1.92	1.53	1.53
Ramachandran outliers (%)	0.38	0.38	0.38	0.38	0.38	0.38	0.38	0.38	0.38	0.38	0.38	0.38
Rotamer outliers (%)	0.93	0.93	0.93	0.93	0.93	0.93	0.93	0.93	0.93	0.93	0.93	0.93
Clashscore	2.18	2.19	1.94	2.19	2.90	2.40	1.93	2.66	2.66	3.13	3.13	2.16
Average *B*-factor (Å²)	25.33	30.86	46.13	48.68	29.57	29.89	29.35	30.82	29.80	28.12	28.39	29.73
macromolecules (Å²)	23.52	29.06	45.40	48.21	28.19	28.71	27.93	29.50	28.56	26.78	27.14	28.49
ligands (Å²)	35.25	31.64	44.36	48.68	26.30	23.59	23.89	25.53	23.36	23.09	22.97	22.17
solvent (Å²)	39.30	45.23	53.37	53.39	41.83	40.44	41.83	42.99	41.18	40.51	39.99	41.24
MolProbity score	0.84	0.91	0.87	0.88	1.09	0.67	0.86	0.92	0.99	1.06	0.92	0.96

Statistics for the highest-resolution shell are shown in parentheses.

Supplementary Table 3: Occupancies of BAB and GBE at the respective delay times when mixing with boric acid (BA) or glycerol (GOL). The BAB occupancy increases with longer delay times after mixing with boric acid while it decreases during mixing with glycerol, as it is esterified to GBE. The total boron content (BAB+GBE) continues to increase along mixing with glycerol due to enhanced inhibitor affinity.

	occupancy	
delay time	BAB	GBE
BA - 0 ms	0 %	0 %
BA - 50 ms	0 %	0 %
BA - 80 ms	35 %	0 %
BA - 100 ms	51 %	0 %
BA - 150 ms	42 %	0 %
BA - 250 ms	49 %	0 %
BA - 500 ms	36 %	0 %
BA - 2000 ms	45 %	0 %
BA - 5000 ms	44 %	0 %
BA - 10000 ms	53 %	0 %
BA - 1 h	57%	0 %

	occupancy	
delay time	BAB	GBE
GOL - 0 ms	57 %	0 %
GOL - 50 ms	55 %	26 %
GOL - 80 ms	57 %	30 %
GOL - 100 ms	57 %	33 %
GOL - 150 ms	35 %	51 %
GOL - 250 ms	29 %	59 %
GOL - 500 ms	33 %	52 %
GOL - 750 ms	26 %	65 %
GOL - 1250 ms	21 %	71 %
GOL - 2500 ms	29 %	57 %
GOL - 5000 ms	25 %	70 %
GOL - 10000 ms	21 %	67 %

BA – boric acid mixing;

GOL – glycerol mixing, presoaked with boric acid;

BAB – boric acid bound;

GBE – glycerol boronic acid ester

Supplementary References

1 Patel, M. P. *et al.* The drug-resistant variant P167S expands the substrate profile of CTX-M β-lactamases for oxyimino-cephalosporin antibiotics by enlarging the active site upon acylation. *Biochemistry* **56**, 3443-3453 (2017).

2 Pemberton, O. A., Tsivkovski, R., Totrov, M., Lomovskaya, O. & Chen, Y. Structural basis and binding kinetics of vaborbactam in class A β-lactamase inhibition. *Antimicrobial agents and chemotherapy* **64**, e00398-00320 (2020).

3 Liu, B. *et al.* Discovery of Taniborbactam (VNRX-5133): A Broad-Spectrum Serine- and Metallo-β-lactamase Inhibitor for Carbapenem-Resistant Bacterial Infections. *Journal of Medicinal Chemistry* **63**, 2789-2801 (2020).

4 Perbandt, M. *et al.* Structural basis to repurpose boron-based proteasome inhibitors Bortezomib and Ixazomib as β-lactamase inhibitors. *Sci Rep* **12**, 1-12 (2022).

5 Lerner, M. & Carlson, H. APBS plugin for PyMOL. *Ann Arbor: University of Michigan* **522** (2006).

6 Jurrus, E. *et al.* Improvements to the APBS biomolecular solvation software suite. *Protein Science* **27**, 112-128 (2018).
